# Caper Bush ( *Capparis spinosa* L.) Minerals and Trace Elements Composition as Affected by Harsh Habitats in Gypsum and Non‐Gypsum Drylands

**DOI:** 10.1002/fsn3.70755

**Published:** 2025-09-18

**Authors:** Elham Yousefi, Mehdi Abedi, Tahereh A. Aghajanzadeh, Diego A. Moreno

**Affiliations:** ^1^ Department of Range Management, Faculty of Natural Resources and Marine Sciences Tarbiat Modares University Noor Mazandaran Province Iran; ^2^ Department of Plant Sciences, Faculty of Science University of Mazandaran Babolsar Iran; ^3^ Laboratorio de Fitoquímica y Alimentos Saludables (LabFAS) CEBAS, CSIC, Campus Universitario de Espinardo −25 Murcia Spain

**Keywords:** caper bush, gypsovag, harsh environments, mineral nutrients, plant‐based foods, sulfur

## Abstract

*Capparis spinosa* L. shrubs (Caper bush) are known for tolerating different ecological conditions. There is a lack of knowledge on the relations between the mineral nutrient composition of the Caper bush aerial plant parts according to the soil conditions where they have grown. Therefore, the aim is to study the mineral composition of soils, fruits, and leaves of *C. spinosa* species grown in two habitats. The samples (soils, leaves, and fruits) of the Caper bush were collected in Abkenar rangeland. Mineral nutrients and trace elements were determined in the samples of soils, fruits, and leaves, using the ICP‐MS technique. The soil samples showed a significant increase in C, Ca, Mg, and S, and a notable decrease in P in the gypsum soils. However, there were no significant differences in the mineral nutrients of the leaves between the two sites, but among trace elements, gypsum‐grown leaves had higher levels of Li, Se, Mo, Cr, and Sr. Apart from nitrogen, all other elements were within the normal range of sufficiency for the plant. For fruit elemental analysis, it was observed that the amount of S, Sr, and Mo was significantly higher and K significantly lower in gypsum habitats. As for fruit essential minerals, there was a slight deficiency in P, Ca, Mg, and Mn, while the levels of other elements were at the normal range. The biochemical adaptation of *C. spinosa* buffered or avoided the excessive accumulation of elements in the different soils. Also, the contents in the leaves and fruits were mainly in the normal range and not accumulating toxic elements.

## Graphical abstract

Elemental comparison of leaves and fruits in gypsum soil showed that gypsum soils had good quality with high levels of Ca, S, and Mg. The mineral composition of Caper bush ( *Capparis spinosa* L.) leaves and fruits was only slightly affected by increasing gypsum in the soil. Additionally, the biochemical adaptation of *C. spinosa* helped buffer or prevent the excessive accumulation of elements in different soils. The contents in the leaves and fruits mainly remained within normal ranges and did not accumulate toxic elements.

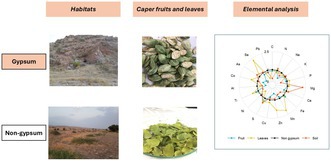

## 1 Introduction

Plants grow in arid and semi‐arid regions, using various strategies to adapt to harsh climatic and soil characteristics, with elements in the soil playing a significant role in plant adaptations (Lambers and Oliveira 2019). Plants require numerous macro and trace minerals to complete their life cycle and maintain healthy growth, which are essential for biological functions (Maillard et al. 2016). The nutritional status of the plant is closely related to the element compositions in soils (White and Brown 2010; Baxter and Dilkes 2012). For instance, plants in calcareous soils have more Al and P elements in their leaves (Cera et al. 2022), whereas in saline soils, the concentration of Na, P, Ca, S, and magnesium elements is high in the leaves (Matinzadeh et al. 2019; Song et al. 2021). Additionally, gypsum soils also contain large amounts of gypsum (CaSO_4_2H_2_O) and are distinguished by a gypsum content of more than 15%, showing significant amounts of Ca, S, and Mg elements in their growing plants (Palacio et al. 2007, 2014). Plant adaptation to gypsum soils is less well understood than in other arid‐region ecosystems, and researchers have been working hard in recent years to learn more about how these plants adapt to dry environments (Escudero et al. 2015).

Gypsum habitats support significant biodiversity; Iran has one of the world's highest plant diversities in gypsum habitats, which has received little attention (Perez‐Garcia et al. 2018; Merlo et al. 2019). Two main groups of plants growing in gypsum soils include gypsovags, plants that can grow in both gypsum and non‐gypsum, and gypsophiles, species primarily restricted to gypsum soils. Gypsovags mainly exhibit low concentrations of gypsum soil elements such as Ca and S compared to gypsophiles (Merlo et al. 2019). Plants that grow in gypsum soils must adapt to soils that are high in Ca, sulfate, and Mg ions but low in N, P, and K. The combination of high Ca and high sulfate far exceeds the plant's nutritional needs and alters the plant's metabolism, which can be toxic to plants, as well as reducing the availability and absorption of macronutrients like P and K (Boscaiu et al. 2013; Bolukbasi et al. 2016; Cera et al. 2021). Although several studies with comparisons of gypsovags and gypsophiles confirmed the higher S, Ca, and Mg in gypsophiles (Escudero et al. 2015). However, few studies compared gypsovag species in gypsum and non‐gypsum soil, which can show species ability in the accumulation of elements in high concentrations (Cera et al. 2021).

*Capparis spinosa* L. is the most important economic species from Capparidaceae, which grows broadly in different habitats (Khanavi et al. 2020; Rakhimova et al. 2021) and several countries such as Turkey, Spain, and Iran largely produce this species for medicinal and food uses (Tlili et al. 2011; Yousefi et al. 2025), besides high potential for restoration (Sakcali et al. 2008; Ashraf et al. 2018). Different parts of *C. spinosa* , including leaves (Turan et al. 2003; Gull et al. 2015), fruits (Özcan 2005; Inagamov et al. 2021), seeds (Özcan and Akgül 1995; Özcan 2005; Haciseferoğullari et al. 2011; Duman and Özcan 2014) and buds (Aliyazicioglu et al. 2013; Gull et al. 2015) are nutrient‐rich. The fruits of this species, as edible parts for humans, can be used as a potential source of minerals in the diet, in particular as a pickle (Gull et al. 2015). *C. spinosa* is widely collected from natural habitats and a few from agricultural lands (Chedraoui et al. 2017), and in Iran, it is restricted to natural habitats (Khoshsima et al. 2017; Saberi et al. 2022). All the studies mentioned focused solely on the nutritional values of plants, without considering environmental conditions, soil quality, and elements. Thus, it is essential to investigate how variations in soil elemental conditions affect the nutritional values of plants.

The largest populations of *C. spinosa* for economic purposes are located in the Southwest of Zagros in the Kazeron region in Iran, with high content of gypsum in the soil in most localities. The commercialization of these fruits is of socioeconomic relevance in the area and supports the economy of local people. However, although high concentrations of some elements in the gypsum soils affect nutritional conditions of food crops, there is no study (to the best of our knowledge) on the nutritional status of this important species in the different soil conditions present in this particular area of gypsum soils. Therefore, this study aimed to investigate (1) how elemental compositions in *C. spinosa* leaves and fruits change in gypsum and non‐gypsum soils; (2) what the mechanism of *C. spinosa* for element accumulations is; and (3) how the nutritional status of Caper bush in the different soils is from an agri‐food perspective.

## 2 Materials and Methods

### 2.1 Study Area

The sampling sites were located in the Abkenar rangeland (N 29° 27′ 22.722″, E 51° 45′ 37.536″; Kazerun city, Fars province, Iran). Two sites with similar climatic and topographic conditions, including gypsum and non‐gypsum sites, were chosen, with the gypsum site being a relict gypsum mine. The average altitude was 768 m a.s.l. The average annual precipitation and average annual temperature were 346 mm and 23°C, respectively, and the climate of the region is defined as semi‐arid according to the Emberger method (Daget 1977).

### 2.2 Soils, Fruits and Leaves Samplings

Three replications of soil samples were taken at random from both sites under the *C. spinosa* canopy in the summer season (August) from a depth of 0–20 cm, transported to the laboratory, and stored at 4°C (in less than 24 h). These soil samples were taken from the same plants that provided leaf and fruit samples. Following soil sampling, the samples were taken to the laboratory and dried in the open air for 2 weeks. To remove lumps and coarse and extra materials such as plant roots, dried soil samples were passed through a 2 mm sieve. The soft soil was then poured into the nylon, which was then ready for measurement. To determine the chemical characteristics, the leaves and fruits were collected, washed, and dried with a paper towel and placed for 24 h at a temperature of 25°C to dry (Minden et al. 2012). Then, the leaves and fruits were dried using a freeze‐drier at −80°C and ground.

### 2.3 Soil Chemical Parameters

After harvesting the soil from under the canopy, the chemical parameters of the soil were determined. Weight comparisons of samples dried at 70°C and 90°C were used to calculate the percentage of soil gypsum (Porta 1998). The pH and electrical conductivity (EC) values were measured in a 1:2.5 soil using an Orion Ionalyzer Model 901 pH meter and an Orion Ionalyzer Model 901 EC meter (Kooch et al. 2022).

### 2.4 Mineral Element Analysis of Leaves, Fruits and Soil Samples

ICP‐MS analysis was conducted to assess the concentration of elements (Fe, Mn, Zn, Ni, Cu, Co, Li, Cd, Hg, Si, Se, Ti, Mo, Al, Cr, Sr., As, V, Pb, Na, K, P, Mg, S, Ca) in leaves, fruits, and soil samples using an Agilent 7500c Inductively Coupled Plasma Mass Spectrometer (Agilent Technologies, USA) (Refer to Palacio et al. 2022 for details on the method applied for gypsum habitats). Total carbon content was analyzed using the Walkley–Black method (Walkley and Black 1934), while total nitrogen was quantified using the semi‐micro‐Kjeldahl method (Bremner and Mulvaney 1982) for soil, fruit, and leaf samples.

### 2.5 Statistical Analysis

Normality of data was tested using the Kolmogorov–Smirnov method, and a *T*‐test was used for statistical analysis of plant and soil parameters for two gypsum and non‐gypsum sites. The change ratio was calculated for macro and micro elements, and it was shown using a radar graph and the effect of cation balance in soil and leaves in order to analyze the ratio of element changes between non‐gypsum and gypsum habitats in both soil and plant parts (Reich et al. 2016).

In addition, PCA was used to investigate the relationship between leaves and fruit elements in the two sites, and PERMANOVA multivariate analysis was used to compare treatments. The vegan package's adonis command was used to analyze the Bray‐Curtis distance in the PERMANOVA test. R software version 4.4.2 (R Foundation for Statistical Computing, Vienna, AT) was used to perform all statistical calculations.

## 3 Results

### 3.1 Soil Parameters

The findings indicate a marked difference in soil acidity between the two sites, with the average soil pH measured at 7.63 ± 0.08 in gypsum areas and 7.40 ± 0.03 in non‐gypsum areas (*p* < 0.05; Table 1). However, the gypsum habitat displayed a significantly higher gypsum content, at 20.88% ± 2.51%, compared to only 5.55% ± 1.03% in the non‐gypsum area (*p* ≤ 0.001; Table 1).

**TABLE 1:** Comparison of average soil acidity, EC, gypsum, and elements in two gypsum and non‐gypsum sites (*n* = 3).

Soil parameter	Non‐gypsum	Gypsum	T‐value	*p* value
pH	7.63 ± 0.08	7.40 ± 0.03	**2.62**	**0.04**
(μds.m^(−1))EC	225 ± 0.04	282 ± 0.16	2.29	0.26
Gypsum (%)	5.55 ± 1.03	20.88 ± 2.51	**−5.64**	**0.001**
**Macro elements (%)**				
C	2.16 ± 0.15	0.56 ± 0.30	**−4.706**	**0.018**
N	0.23 ± 0.05	0.37 ± 0.04	2.574	0.066
Na	0.18 ± 0.08	0.12 ± 0.18	2.883	0.0754
K	0.82 ± 0.24	0.88 ± 0.24	1.588	0.188
P	0.14 ± 0.12	0.5 ± 0.05	**−4.929**	**0.024**
Mg	0.58 ± 0.04	1.75 ± 0.36	**3.863**	**0.050**
S	0.030 ± 0.0	0.042 ± 0.02	**6.103**	**0.012**
Ca	4.06 ± 1.06	5.17 ± 1.21	**1.532**	**0.002**
**Micro elements (ppm)**				
Fe	17,660 ± 1278	10,990 ± 1415.30	**−3.494**	**0.025**
Mn	756.86 ± 31.87	494.66 ± 40.38	**−5.062**	**0.008**
Zn	78.45 ± 3.37	72.36 ± 17.77	−0.337	0.766
Ni	106.53 ± 7.74	95.03 ± 10.79	−0.866	0.440
Cu	31.83 ± 1.43	27.04 ± 2.56	−1.637	0.196
Co	15.68 ± 1.14	13.93 ± 2.11	−0.730	0.517
Li	21.17 ± 1.053	36 ± 0.01	1.929	0.189
Cd	0.58 ± 0.01	0.49 ± 0.31	0.299	0.793
**Hg**	6.67 ± 0.16	6.25 ± 0.94	0.439	0.701
Si	34,214 ± 7312.1	34451.6 ± 4682.16	0.027	0.980
Se	0.64 ± 0.09	0.67 ± 0.10	0.189	0.859
Ti	2869.21 ± 174.7	1686.37 ± 287.9	**−3.512**	**0.0337**
Mo	4.58 ± 0.07	6.22 ± 1.25	1.309	0.32
Al	4775.48 ± 459.07	2749.26 ± 786.32	−2.225	0.106
Cr	115.19 ± 7.27	81.80 ± 11.15	−2.509	0.076
Sr	121 ± 7.5	3601 ± 1712.4	2.032	0.179
As	8.20 ± 0.51	10.28 ± 1.31	1.475	0.250
V	92.75 ± 6.1	72.79 ± 9.60	−1.753	0.167
Pb	17.41 ± 0.29	13.92 ± 2.14	−1.619	0.243

*Note:* Data represent the mean (± SE) of three measurements with soil. Relative responses and significance are shown in Table 3. Bold values indicate significant differences between gypsum and nin‐gypsum at 0.05.

Elemental analyses revealed a notable increase in sulfur (0.042% ± 0.02% in gypsum and 0.030% ± 0.0%; *p* < 0.05), magnesium (1.75% ± 0.36% in gypsum compared to 0.58% ± 0.04% in non‐gypsum; *p* < 0.05), and calcium (5.17% ± 1.21% in gypsum vs. 4.06% ± 1.06% in non‐gypsum; *p* < 0.01) in gypsum soil when contrasted with non‐gypsum soil. Conversely, phosphorus levels were significantly lower in gypsum soil (0.05% ± 0.05% in gypsum compared to 0.14% ± 0.12% in non‐gypsum; *p* < 0.05; Table 1), as well as carbon (0.56% ± 0.30% in gypsum vs. 2.16% ± 0.15% in non‐gypsum; *p* < 0.05). In terms of trace elements, higher concentrations were observed in non‐gypsum soil for Fe (10,990 ± 1415.30 ppm in gypsum vs. 17,660 ± 1278 ppm in non‐gypsum; *p* < 0.05), Ti (1686.37 ± 287.9 ppm in gypsum against 2869.21 ± 174.7 ppm in non‐gypsum; *p* < 0.05), and Mn (494.66 ± 40.38 ppm in gypsum compared to 756.86 ± 31.87 ppm in non‐gypsum; *p* < 0.05) (Table 1).

### 3.2 Caper Bush Mineral Elements

The assessment of the mineral content in the leaves showed no significant differences in essential minerals or macro elements (Table 2). Conversely, trace elements exhibited significant variations, with Li (3.70 ± 0.23 ppm in gypsum vs. 1.66 ± 0.38 ppm in non‐gypsum; *p* < 0.05), Se (1.84 ± 0.23 ppm in gypsum vs. 0.80 ± 0.04 ppm in non‐gypsum; *p* < 0.05), Mo (5.59 ± 0.57 ppm in gypsum vs. 2.58 ± 0.44 ppm in non‐gypsum; *p* < 0.05), Cr (8.08 ± 0.4 ppm in gypsum vs. 3.57 ± 0.10 ppm in non‐gypsum; *p* < 0.01), and Sr (693 ± 50.89 ppm in gypsum vs. 175.04 ± 10.71 ppm in non‐gypsum; *p* < 0.01) showing higher concentrations in leaves collected from the gypsum site (Table 2).

**TABLE 2:** The comparisons between two gypsum and non‐gypsum sites on high (%) and low (ppm) consumption nutrient content of leaves and fruits of *C. spinosa* (*n* = 3).

Elements	Plant parts	Non‐gypsum	Gypsum	T‐value	*p* value
**Macro elements (%)**					
C	Leaf	41.27 ± 0.90	40.06 ± 0.10	−1.338	0.310
	Fruit	46.70 ± 0.16	47.24 ± 0.12	2.678	**0.059**
N	Leaf	1.84 ± 0.17	1.50 ± 0.23	−1.178	0.310
	Fruit	3.58 ± 0.28	3.43 ± 0.04	−0.554	0.633
Na	Leaf	0.06 ± 0.10	0.052 ± 0.14	−0.332	0.758
	Fruit	0.02 ± 0.00	0.01 ± 0.00	−2.773	0.108
K	Leaf	2.38 ± 2.60	2.23 ± 3.57	0.354	0.743
	Fruit	1.11 ± 0.02	1.01 ± 0.00	**−4.651**	**0.020**
P	Leaf	0.15 ± 0.16	0.18 ± 0.08	1.628	0.208
	Fruit	0.09 ± 0.00	0.08 ± 0.00	−2.191	0.149
Mg	Leaf	1.12 ± 0.90	1.24 ± 2.24	0.520	0.643
	Fruit	0.14 ± 0.00	0.14 ± 0.00	−0.247	0.817
Ca	Leaf	3.13 ± 1.37	3.37 ± 3.33	0.252	0.819
	Fruit	0.32 ± 0.02	0.25 ± 0.03	−1.930	0.130
S	Leaf	0.03 ± 0.06	0.04 ± 0.01	1.048	0.402
	Fruit	0.64 ± 0.02	0.80 ± 0.00	**10.356**	**0.005**
Micro Element (ppm)					
Fe	Leaf	310 ± 20	332 ± 20	0.684	0.532
	Fruit	167.21 ± 37.65	199.60 ± 35.88	0.623	0.567
Mn	Leaf	71.80 ± 11.5	146.26 ± 53.6	1.358	0.298
	Fruit	14.22 ± 0.76	12.02 ± 0.08	−2.872	0.101
Zn	Leaf	15.19 ± 0.42	28.38 ± 4.57	2.874	0.101
	Fruit	132.450 ± 12.06	59.634 ± 15.54	−0.847	0.467
Ni	Leaf	15.13 ± 4.81	8.35 ± 1.90	−1.313	0.293
	Fruit	4.70 ± 0.41	4.10 ± 0.00	−1.461	0.281
Cu	Leaf	8.24 ± 0.58	9.90 ± 1.62	0.968	0.417
	Fruit	14.24 ± 1.39	10.33 ± 1.03	−2.263	0.092
Co	Leaf	0.27 ± 0.05	0.42 ± 0.09	1.471	0.232
	Fruit	0.34 ± 0.12	0.18 ± 0.04	−1.320	0.300
Li	Leaf	1.66 ± 0.38	3.70 ± 0.23	**4.565**	**0.016**
	Fruit	0.19 ± 0.05	0.24 ± 0.07	0.190	0.860
Cd	Leaf	0.56 ± 0.03	0.65 ± 0.12	0.719	0.538
	Fruit	0.12 ± 0.01	0.12 ± 00	−0.769	0.517
Hg	Leaf	8.79 ± 0.99	7.21 ± 0.63	1.343	0.262
	Fruit	0.54 ± 0.25	1.34 ± 0.70	0.863	0.459
Si	Leaf	26691.60 ± 5786.13	42252.8 ± 4784.2	2.073	0.109
	Fruit	448.86 ± 36.44	372.43 ± 132.91	−0.554	0.629
Se	Leaf	0.80 ± 0.04	1.84 ± 0.23	**4.440**	**0.041**
	Fruit	1.74 ± 0.76	1.40 ± 0.60	−0.344	0.749
Ti	Leaf	19.16 ± 2.7	31.04 ± 3.7	2.571	0.068
	Fruit	2.75 ± 0.82	1.16 ± 0.03	−1.942	0.191
Mo	Leaf	2.58 ± 0.44	5.59 ± 0.57	**4.179**	**0.016**
	Fruit	0.47 ± 0.03	1.32 ± 0.07	**10.989**	**0.002**
Al	Leaf	309.73 ± 75.6	215.75 ± 38.40	−1.108	0.349
	Fruit	40.57 ± 13.67	11.34 ± 1.09	−2.1312	0.165
Cr	Leaf	3.57 ± 0.10	8.08 ± 0.4	**11.29**	**0.005**
	Fruit	0.48 ± 0.12	2.93 ± 0.67	3.615	0.062
Sr	Leaf	175.04 ± 10.71	693 ± 50.89	**9.966**	**0.007**
	Fruit	21.07 ± 0.65	42.53 ± 3.03	**6.929**	**0.015**
As	Leaf	0.61 ± 0.02	0.59 ± 0.07	0.266	0.811
	Fruit	1.10 ± 0.41	0.35 ± 0.00	−1.859	0.203
V	Leaf	0.82 ± 0.09	1.12 ± 0.09	2.284	0.085
	Fruit	—	—	—	—
Pb	Leaf	14.66 ± 3.78	4.52 ± 1.84	−2.413	0.098
	Fruit	1.29 ± 0.10	1.10 ± 0.06	−1.575	0.208

*Note:* Data represent the mean (± SE) of three measurements with leaves and fruits. Relative responses and significance are shown in Table 3. Bold values indicate significant differences between gypsum and nin‐gypsum at 0.05.

In the analysis of fruit nutrients, it was found that sulfur (S) levels were significantly higher in the gypsum habitat (0.80% ± 0.00%) compared to the non‐gypsum habitat (0.64% ± 0.02%; *p* < 0.01). Conversely, potassium (K) levels were significantly lower in the gypsum habitat, recording at 1.01% ± 0.00% versus 1.11% ± 0.02% in the non‐gypsum habitat (*p* < 0.05) (Table 2). Furthermore, among essential microelements, strontium (Sr) was notably elevated in gypsum (42.53 ± 3.03 ppm) compared to non‐gypsum (21.07 ± 0.65 ppm; *p* < 0.05), and molybdenum (Mo) levels were also higher in gypsum (1.32 ± 0.07 ppm) compared to non‐gypsum (0.47 ± 0.03 ppm; *p* < 0.05) (Table 2).

**FIGURE 1:**
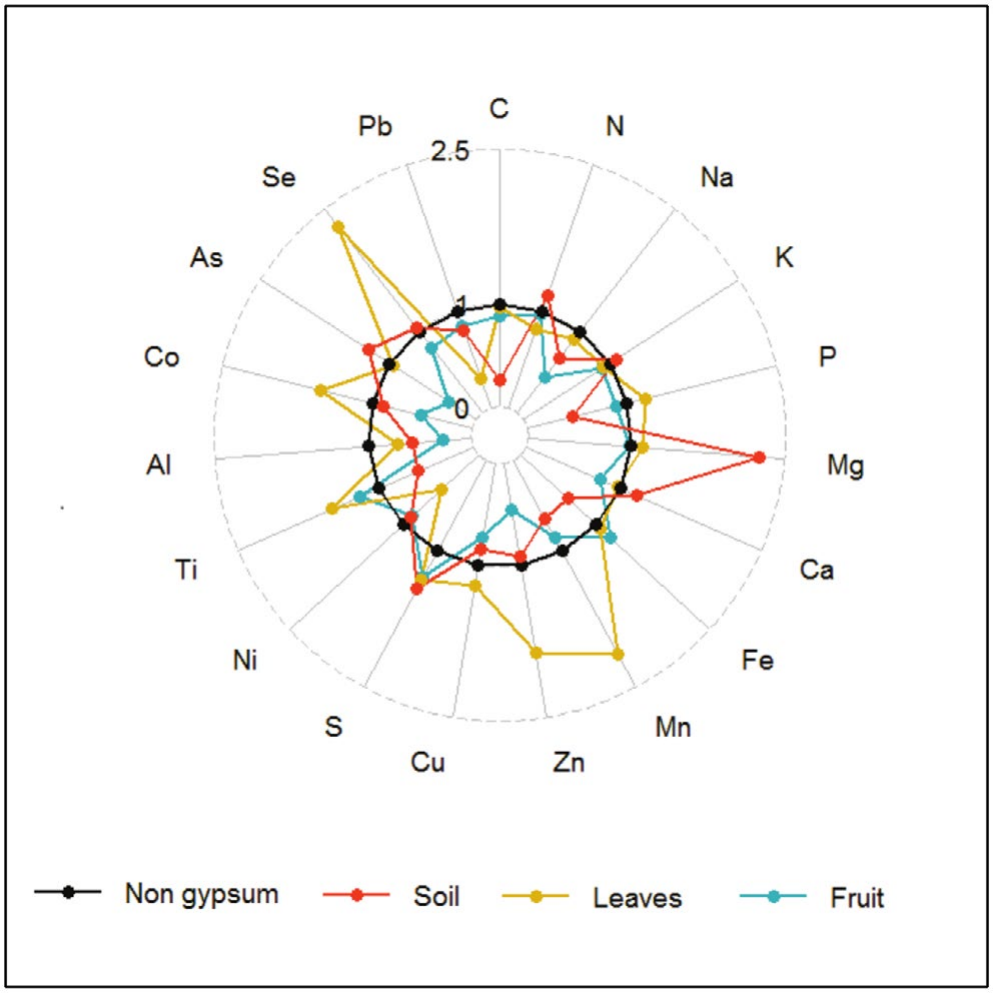
The changes of gypsum site compared to non‐gypsum site on mineral nutrient content in soil, leaves, and fruit of *C. spinosa* . Radar diagrams showing response ratios relative to non‐gypsum conditions: Non‐gypsum (black); soil (orange), leaves (yellow), and fruit (blue). Sr, Mo, and Cr were excluded from this figure due to their extraordinarily large changes. For absolute contents, see Tables 1 and 2.

**FIGURE 2:**
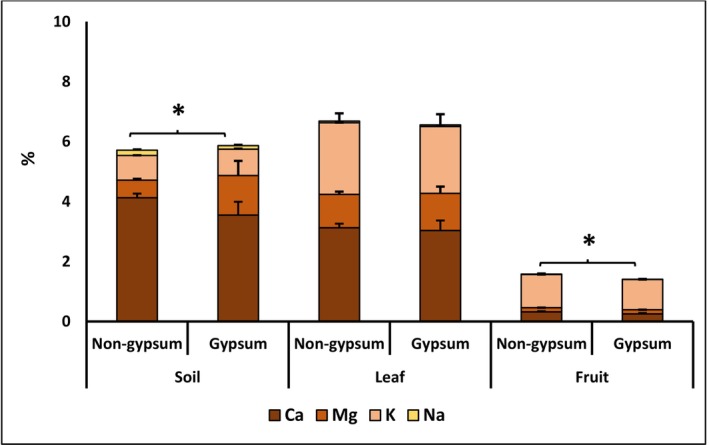
The impact of gypsum site variations on the cation balance in the soil, leaves, and fruits of *C. spinosa*. Data represent the mean (± SE; *n* = 3) of the contents of potassium (K), calcium (Ca), sodium (Na), and magnesium (Mg) in soil, leaf, and fruit triple measurements with three plants each in percentage. * indicates significant differences between the sum of cations of gypsum and non‐gypsum sites for soil, leaves, and fruits using the *T*‐test. The comparisons of each cation were also presented in Tables 1 and 2.

The comparison of element ratios is valuable for assessing different treatments (Reich et al. 2016). In the soil, leaves, and fruits, significant changes were observed between soil types for certain elements (refer to Tables 1 and 2). Notably, there were substantial ratio variations in leaf elements (Li, Cr, Sr, Se and Mo; *p* < 0.05), as well as in Sr and Mo in fruits (*p* < 0.05), with over 100% changes (see Table 3). The radar graph illustrated that N, S, Mg, and Li in the soil, and Mo, Se, Cr, Li, Mn, Zn, and S in leaves exhibited the most pronounced changes. Furthermore, leaves and fruit elements displayed the most positive and negative changes in gypsum soil, respectively (refer to Table 3, Figure 1). It is important to note that the differences in the individual cation contents were less noticeable between the soil and fruits at the two sites, compared to their combined sum, and were not noticeable in both individual and sum cations for leaves (Figure 2).

**TABLE 3:** relative change in gypsum site compared to non‐gypsum site on the content of mineral nutrients in soil, leaf and fruit (*n* = 3).

Elements	Soil	Leaf	Fruit
**C**	−74.1**	−2.9 ns	*−11.7 ns*
**N**	*60.9 ns*	−18.5 ns	−4.20 ns
**Na**	*−32.6 ns*	−10.3 ns	−56.5 ns
**K**	6.6 ns	−6.5 ns	−8.6**
**P**	−53.3**	18.4 ns	−10.9 ns
**Mg**	*−124.9 ns*	11.3 ns	−0.7 ns
**Ca**	17.2***	−2.9 ns	−21.2 ns
**Fe**	−**37.8** *	6.5 ns	19.4 ns
**Mn**	**−35.5** ***	114.3 ns	−15.5 ns
**Zn**	−7.8 ns	86.8 ns	−54.9 ns
**S**	16.1*	18.2 ns	**28.7** ***
**Ni**	−10.8 ns	−44.6 ns	−12.8 ns
**Li**	70.1 ns	**122.2** *	—
**Cu**	−15.1 ns	20.2 ns	−27.5 ns
**Co**	−11.2 ns	52.1 ns	−48.8 ns
**Si**	0.7 ns	58.3 ns	20.5 ns
**V**	−21.5 ns	*35.6 ns*	—
**Ti**	−**41.1** *	*62.0 ns*	−57.8 ns
**Al**	−42.5 ns	−29.0 ns	−72.1 ns
**Cr**	*−29.01 ns*	**136.7** **	510.4 ns
**Sr**	200 ns	**296.1** **	**101.9** **
**Se**	4.1 ns	**128.8** *	−19.5 ns
**As**	25.2 ns	−3.5 ns	−68.2 ns
**Cd**	−15.8 ns	15.7 ns	—
**Hg**	−6.4 ns	−17.9 ns	—
**Mo**	35.9 ns	**116.9** *	**180.9** ***
**Pb**	−20.1 ns	*−69.2 ns*	−14.7 ns

*Note:* Data expressed as relative change in % to non‐gypsum sites. Significant difference from the gypsum site is indicated by bold font Unpaired Student's *t*‐test on original values; ns not significant, italic marginally significant, Relative increase compared to the control (non‐gypsum) is accentuated in orange, relative decrease in blue. Bold values indicate siginifacant differences.

* *p* < 0.05.

** *p* < 0.01.

*** *p* < 0.001.

According to Kalra (1997), the nutrient values for nitrogen in leaves (Table 2) were deficient, whereas the levels of potassium, magnesium, and calcium were within the normal range. The content of essential microelements, such as copper, iron, manganese, molybdenum, and zinc, in the leaves was also within the normal range of nutrient efficiency (Table 2) (Kalra 1997). Furthermore, as noted by Kalra (1997), there were slight deficiencies in the macro elements phosphorus (P), calcium (Ca), and magnesium (Mg) in fruits, whereas the nitrogen (N), sulfur (S), and potassium (K) levels were normal (see Table 2). Regarding micronutrients, manganese (Mn) showed a deficiency, but the levels of copper (Cu), zinc (Zn), iron (Fe), and molybdenum (Mo) remained within the normal range (Table 2) (Kalra 1997).

**FIGURE 3:**
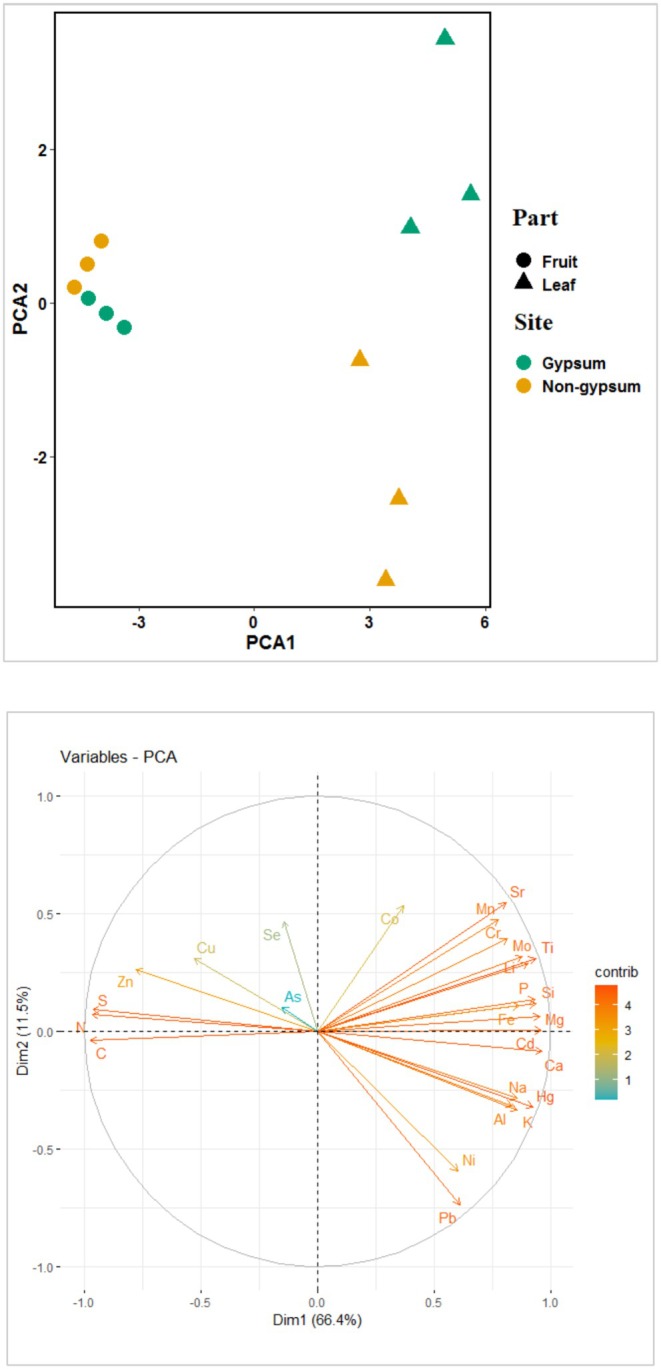
Principal component analysis results of the leaves and fruits in two gypsum and non‐gypsum sites.

To compare the treatments, we conducted a PCA analysis (Figure 3). The first axis accounted for 66.4% of the variations, which corresponded to the majority of highly contributing elements, excluding Na, Cu, Co, As, and Se. The second axis explained 11.5% of the variations, relating only to the elements Ni and Pb (Table 4). Additionally, the results of the PERMANOVA test indicated a significant effect of plant parts (F = 8.72 and *p* > 0.01), while the effects of sites and their interactions showed marginal significance (See Figure 3 and Table 5).

**TABLE 4:** The percentage of contributions of elements in the first and second axes of PCA.

Elements	First axis	Second axis
N	5.39***	0.17
C	5.48***	0.05
Na	4.27	2.72
K	4.25***	3.72
Ca	5.39***	0.25
Mg	5.28***	0.14
Li	4.74***	2.74
Al	4.05***	3.36
P	5.04***	0.62
Si	5.10***	0.47
Ti	5.07***	3.21
Cr	3.84***	5.15
Mn	3.48***	7.53
Fe	4.34***	0.39
Co	0.78	9.49
Ni	2.09***	11.72***
Cu	1.61	3.16
Zn	3.49***	2.27
As	0.13	0.35
Se	0.12	7.30
S	5.36***	0.28
Sr	3.79***	9.93
Mo	4.46***	3.35
Cd	5.33***	0.00
Hg	4.96***	3.45
Pb	2.17***	18.16***

*Note:* Bold values indicate siginifacant differences.

*** indicate siginificance at < 0.001.

**TABLE 5:** Examining the significant effects of the elements in the four studied treatments using the PERMANOVA.

Treatments	Df	Sum of Sq.	F	*R* ^2^	*p* value
Part	1	184.51	8.72	0.645	**0.001**
Site	1	22.04	3.14	0.077	*0.072*
Part × Site	1	23.27	3.31	0.081	*0.061*

*Note:* Bold values indicate siginifacant differences.

## 4 Discussion

Species adapted to harsh drylands on gypsum soils show biochemical adaptations that help them function and offer resilient food security alternatives amid global warming and food shortages. Findings indicate that, despite significant differences in soil elements, the contents of major elements in the leaves and fruits of *C. spinosa* from non‐gypsum and gypsum soils were only slightly different. Unlike gypsophiles, which can accumulate dominant elements in gypsum soils (Escudero et al. 2015), *C. spinosa* does not allow these accumulations. Most nutrients in the leaves and fruits were at normal levels, with no toxic concentrations for the plant or for consumption (Kalra 1997).

### 4.1 Gypsum Soils Had Good Quality and High Content of Ca, S and Mg

This study confirms our first hypothesis, showing significant increases in calcium, manganese, and sulfur, alongside decreases in phosphorus, carbon, iron, and titanium (*p* < 0.05). These results align with prior research indicating greater abundance of calcium, magnesium, and sulfur in gypsum soils (Escudero et al. 1999; Ruiz et al. 2003; Moore et al. 2014). They also reflect findings that gypsum habitats have less organic matter (Boscaiu et al. 2013) and support previous studies on trace elements in gypsum soil (Cera et al. 2021). Thus, gypsum soils present harsh conditions for species establishment; however, the high levels of carbon and calcium suggest these soils can still support plant growth.

### 4.2 Caper Bush Leaf and Fruit Mineral Composition Was Slightly Affected by Increasing Gypsum in Soil

The findings of the study, supporting our second hypothesis, reveal an increase in S, Sr., and Mo in the fruits from gypsum soil, with no significant differences in macro element levels—namely, Ca, Mg, and S—in leaves from two different sites. This observation aligns with our earlier research, which noted rising sulfur compounds of fruits in gypsum soil (Yousefi et al. 2025). It contrasts with earlier studies suggesting higher levels of Ca, S, K, and Li, and lower P in gypsovags leaves (Cera et al. 2021; Robson et al. 2017). The lack of significant differences in Ca, Mg, S, and P may stem from the lower gypsum content (20%) in our study compared to 70% in Cera et al. (2021). Moreover, the noticeable increases in Li, Se, Mo, Cr, and Sr in leaves from gypsum soil correspond with findings from previous studies that documented higher levels of Li and Cr (Cera et al. 2021) and Sr (Lu and Meyers 2003; Merlo et al. 2019). The low levels of microelements make it difficult to fully understand their complex roles in how plants adapt to gypsum soil (Merlo et al. 2019).

### 4.3 Caper Bush Leaves and Fruits Did Not Accumulate Toxic Elements

This study confirmed our third hypothesis, finding that nutrient levels in leaves and fruits were normal, without reaching toxic levels at both sites. This shows that *C. spinosa* fruit is nutrient‐rich. Higher microelements and heavy metals in gypsum soil did not influence their accumulation in fruit and leaves. These findings support the potential application of *C. spinosa* leaves and fruits from challenging environments, such as gypsum soil, in the food industry. This aligns with earlier discoveries of high antioxidant capacity and phenolic compounds in severe conditions (Yousefi et al. 2025). *C. spinosa* fruits are high in potassium and low in sodium. Özcan and Akgül (1995) and Özcan (2005) indicated that *Capparis* fruits have high potassium and very low sodium, along with low trace elements beneficial for food products. These findings align with other studies suggesting *C. spinosa* fruits and seeds are nutrient‐rich with low trace elements. Inagamov et al. (2021) reported microelements like Fe, Zn, Cu, Mn, and Se, consistent with our findings. Haciseferoğullari et al. (2011) and Duman and Özcan (2014) noted that *Capparis* seeds are rich in macro (K, Ca, Mg, Na, P) and microelements (Zn, Cu, Fe). In conclusion, caper seeds offer a mineral‐rich source beneficial for humans and livestock, providing essential nutrients for metabolic functions.

## 5 Conclusions

Gypsovag species can grow in both gypsum and non‐gypsum habitats. *C. spinosa* (caper bush) shifted its leaf elemental compositions according to nutrient soil availability. Although there were significant changes in soil macro elements, there were no significant differences in leaf macro elements. This result supports the fact that gypsovags are not adapted to accumulate Ca or S, and increasing their amount in the plant parts in gypsum soil depends on the gypsum soil content and other environmental conditions (e.g., water availability, biotic stress). No significant differences between leaves and fruits of both sites suggest that this species grows well in gypsum and non‐gypsum soils. High nutrient values along with low trace metals also show this species could be used broadly in the restoration of harsh lands for agri‐food production as well as for sustainable food security in the context of global warming, which would affect cropping areas worldwide.

## Author Contributions

**Elham Yousefi:** investigation (equal), resources (equal), writing – original draft (equal). **Mehdi Abedi:** conceptualization (equal), project administration (equal), resources (equal), supervision (equal), validation (equal), writing – review and editing (equal). **Tahereh A. Aghajanzadeh:** methodology (equal), validation (equal), writing – review and editing (equal). **Diego A. Moreno:** conceptualization (equal), project administration (equal), supervision (equal), writing – review and editing (equal).

## Ethics Statement

This study does not involve any human or animal testing.

## Consent

Written informed consent was obtained from all study participants.

## Conflicts of Interest

The authors declare no conflicts of interest.

## Data Availability

Data will be made available on request.
